# An Unusual Presentation of Apical Hypertrophic Cardiomyopathy in an Orthotopic Heart Transplant Recipient

**DOI:** 10.7759/cureus.44344

**Published:** 2023-08-29

**Authors:** Victor H Molina-Lopez, Andrew Engel-Rodriguez, Porfirio E Diaz-Rodriguez, Sonia Vicenty-Rivera

**Affiliations:** 1 Cardiology, Veterans Affairs Medical Center, San Juan, PRI; 2 Internal Medicine, Veterans Affairs Medical Center, San Juan, PRI

**Keywords:** mid-ventricular outflow tract obstruction, orthotopic heart transplant, left ventricular apical hypertrophy, apical hypertrophic cardiomyopathy, hypertrophic cardiomyopathy

## Abstract

In this case study, we present the evaluation of an orthotopic heart transplant (OHT) patient who presented with persistent shortness of breath and dizziness upon standing. The investigation uncovered the presence of progressive hypertrophic cardiomyopathy (HCM) in the transplanted heart, a condition first detected 11 years after the transplantation. Utilizing echocardiography with global longitudinal strain (GLS), we determined that the HCM likely originated from genetic predominance inherited from the heart donor rather than hypertensive disease. This finding highlights the significance of genetic factors in post-transplant complications and warrants further investigation into the long-term effects of heart transplantation on recipient health.

## Introduction

Left ventricular apical hypertrophic cardiomyopathy (ApHCM) is a rare phenotypic variant of hypertrophic cardiomyopathy (HCM). It was initially identified in Japan in 1976 and has since gained growing recognition [1]. ApHCM is characterized by localized hypertrophy in the apical segments of the left ventricle (LV). Specific ECG findings, notably deep T-wave inversions in the precordial leads, aid in its diagnosis. Imaging studies, such as echocardiography and cardiac magnetic resonance imaging (cMRI), reveal a characteristic spade-shaped appearance of the apical cavity in diastole, with hypertrophy predominantly affecting apical segments [2]. The prevalence of ApHCM varies across populations, with reported incidences ranging from 1% to 2% in Western countries [3]. This case report presents a patient who underwent an orthotopic heart transplant (OHT) and experienced a stable post-transplant course; however, he had progressive left ventricular hypertrophy and was eventually diagnosed with ApHCM 16 years after the OHT. Cases of ApHCM in OHT are a unique occurrence in the medical literature. Addressing them requires specialized clinical approaches due to the limited established therapies [4-5].

## Case presentation

A 75-year-old male was evaluated for shortness of breath and dizziness upon standing from a sitting position. The patient denied experiencing chest pain, orthopnea, paroxysmal nocturnal dyspnea, syncope episodes, or palpitations. He was an active smoker with a smoking history of more than 90 pack years and had chronic obstructive pulmonary disease, chronic renal disease stage IIIa, type II diabetes mellitus for over 30 years, and hypertension and was an OHT recipient on chronic immunosuppressive therapy. The OHT was performed 23 years ago to treat end-stage heart failure from dilated cardiomyopathy secondary to viral disease after he required five months of a left ventricular assist device as a bridge to transplantation. The cardiac allograft was from an 18-year-old donor. He meticulously complied with medical and immunosuppressive therapy over the past 23 years. The patient's family history was remarkable for a first-degree relative with dilated cardiomyopathy progressing to end-stage heart failure.

The first instance of mild left ventricular hypertrophy was identified 10 years after OHT on routine transthoracic echocardiogram (TTE). Over the subsequent years, annual follow-up TTE revealed worsening apical left ventricular hypertrophy. Surveillance endomyocardial biopsies were performed up to five years post-transplant, all showing Grade 0 (no rejection), requiring minor adjustments to his immunosuppressive therapy. His medications mainly remained unchanged over the years, including lisinopril, rosuvastatin, finasteride, tamsulosin, levothyroxine, mycophenolate mofetil, prednisone, tacrolimus, and insulin therapy. He denied illicit drug use and alcohol consumption but became an active smoker after OHT. Although he had a history of alcohol abuse, he had been in remission for over 25 years.

**Figure 1:**
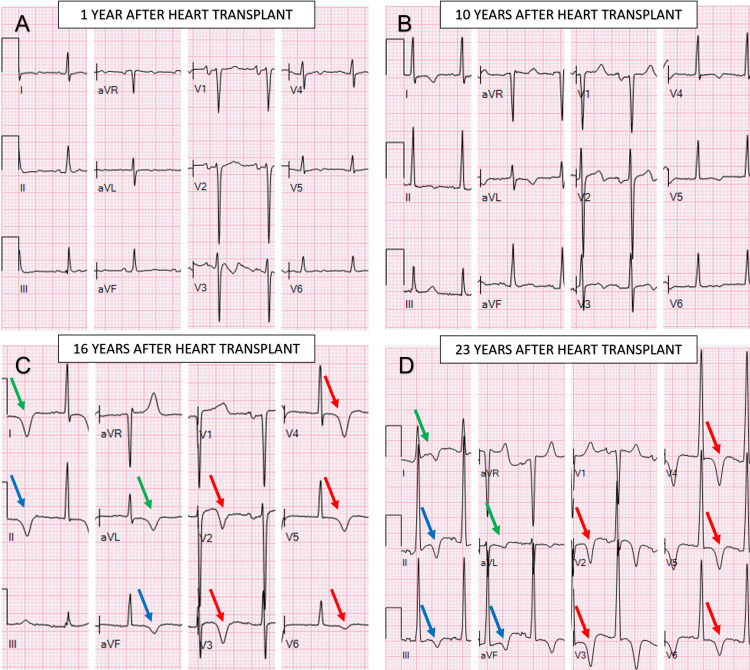
Evolution of ECG left ventricular hypertrophy repolarization changes The first ECG depolarization abnormalities suggestive of hypertrophic disease were seen around 10 years post-transplant. By 16 years post-transplant, the patient exhibited voltage criteria for Left ventricular hypertrophy and deep T-wave inversions typical for ApHCM, which worsened as the condition progressed. Patients with ApHCM are associated with marked T-wave inversions in the precordial leads (red arrows). Inverted T-waves were also observed in the inferior (blue arrows) and high lateral leads (green arrows)

Vital signs were within normal limits. Blood pressure logs demonstrated well-controlled hypertension over time. Overall, his physical exam was unremarkable, and he appeared clinically euvolemic. The cardiac exam revealed a regular rate and rhythm with a prominent apical pulse but no murmurs. No jugular venous distention was evident at 45 degrees, and there was no evidence of hypoxemia on pulse oximetry. The 12-lead ECG demonstrated the criteria for ventricular hypertrophy with T-wave inversions in precordial leads (Figure 1A-1D).

Laboratory parameters were remarkable for stable chronic kidney disease stage IIIb, with no elevated proteins in serum and no anemia. Urinalysis revealed very mild proteinuria in the setting of chronic diabetes mellitus for >30 years. Urine and serum protein electrophoresis showed no abnormal proteins. The baseline NT-pro-BNP level was 3,500 pg/mL. The 24-hour Holter evaluation was unremarkable for atrial or ventricular arrhythmias. The patient had no history of ventricular tachycardia or atrial fibrillation.

**Figure 2:**
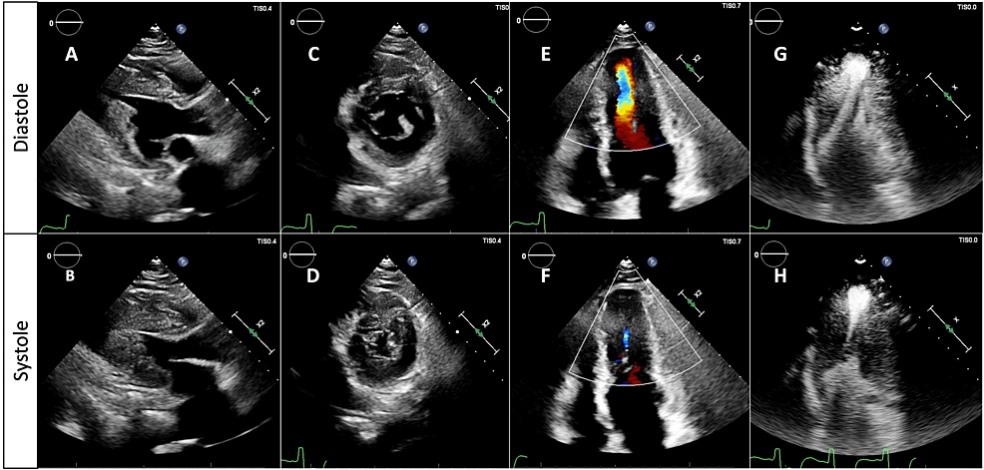
Echocardiographic evaluation 22 years after the OHT TTE follow-up 22 years after the OHT with severe ApHCM demonstrated in (A-B) PLAX, (C-D) parasternal short axis at the level of the papillary muscles, and (E-F) apical 4 chamber view with color flow Doppler. (G-H) The contrast-enhanced TTE in apical 2 chamber view shows severe apical left ventricular hypertrophy with the classical “spade-shaped” ventricular cavity in diastole, with mid-cavity obliteration during systole, and an apical aneurysm

TTE revealed a severe left ventricular ApHCM pattern with a left ventricular ejection fraction of >70%, a small left ventricular cavity, and an apical aneurysm (Figure 2A-2H). The diastolic interventricular septal thickness in the parasternal long axis (PLAX) view was 1.3 cm. Contrast-enhanced TTE delineated the apical aneurysm without an associated apical thrombus (Figure 2G-2H).

**Figure 3:**
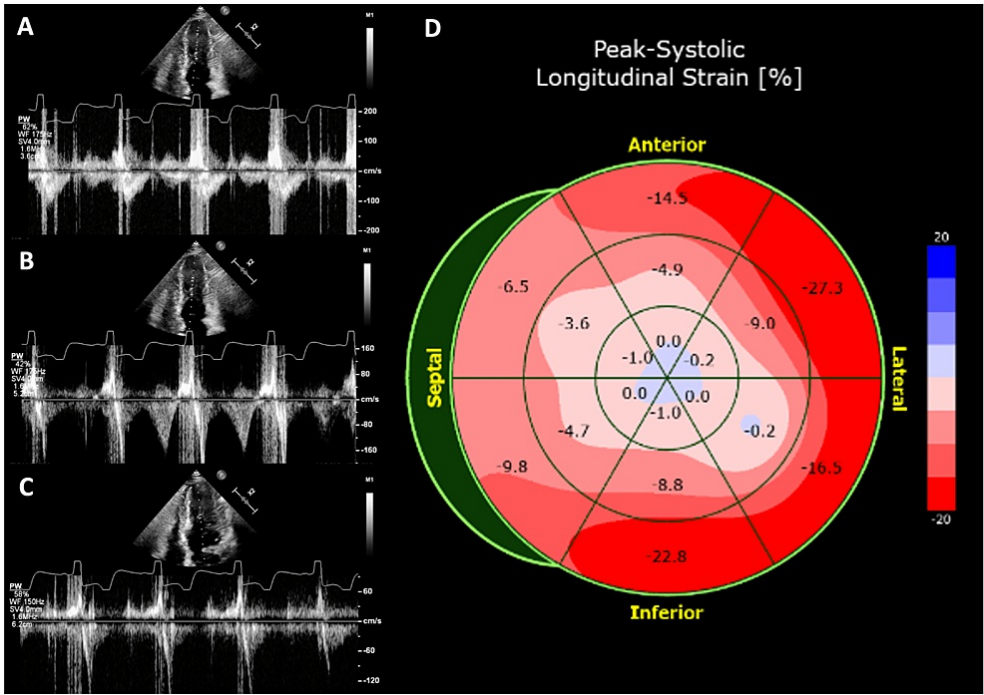
Echocardiographic evaluation 22 years after the OHT demonstrating the midventricular cavity obliteration (A-C) and strain polar plot (D) showing loss of longitudinal strain apically

On TTE evaluation, there was no left ventricular outflow tract (LVOT) obstruction, but mid-ventricular systolic collapse was present (Figure 3A-3C). There was no abnormal systolic motion of the anterior mitral leaflet. Strain analysis revealed a reduced global longitudinal strain (GLS) of -6.9% with a strain polar plot showing loss of longitudinal strain apically (Figure 3D).

**Figure 4:**
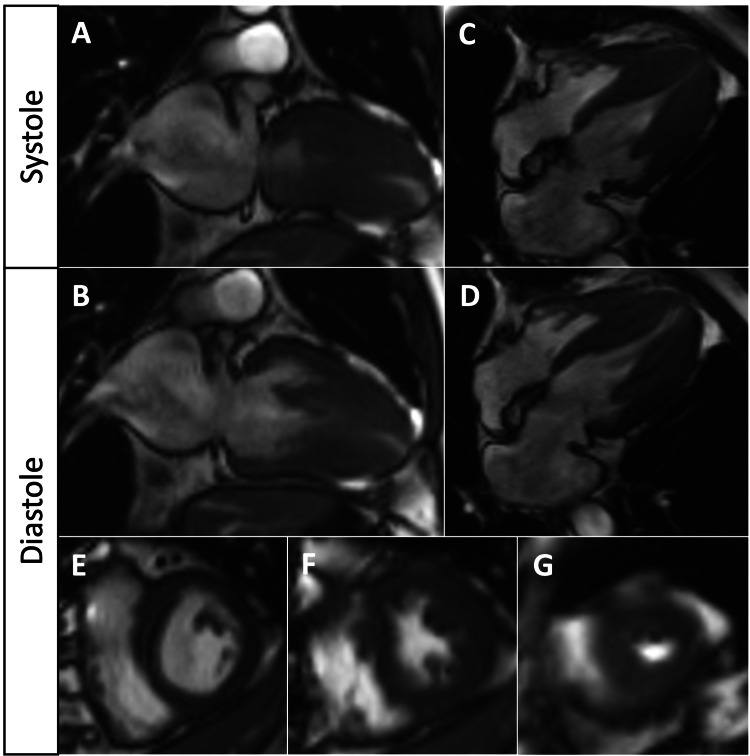
cMRI revealing ApHCM with apical aneurysm formation Severe ApHCM in long‐axis two‐chamber views (A-B) and long‐axis four‐chamber views (C-D) with mid-ventricular obstruction and ventricular cavity obliteration during systole. Short axis views (E-G) demonstrate the sparing of basal segments with apical hypertrophy, predominantly affecting mid and apical segments with a small apical aneurysm (G)

To further assess the hypertrophy as indicated by the TTE findings, a cMRI was conducted (Figure 4). Measurements of the left ventricular cavity at end-diastole revealed dimensions of 1.3 cm at the basal septal wall and 0.8 cm at the basal lateral free wall. At the mid-cavity, dimensions were 1.8 cm at the septal wall and 1.6 cm at the lateral free wall, while at the apical segment, both the septal wall and the lateral free wall measured 1.5 cm. During systole, the septum and lateral free wall converged, resulting in complete obliteration of the lumen at the mid-cavity level. An apical aneurysm was identified with associated cortical thinning at the apex. The cMRI showed no evidence of a flow void or stenotic jet at the LVOT and no irregular systolic motion of the anterior mitral leaflet.

Given the progressive nature of ApHCM, characterized by mid-cavity obliteration, the patient was started on beta-blocker therapy and adjusted to the maximum tolerable dose based on symptoms. He had no prior episodes of atrial fibrillation or ventricular tachyarrhythmias as evidenced by ambulatory electrocardiographic monitoring. Due to his age, comorbidities, and care goals, he was considered ineligible for reevaluation for a subsequent transplant. Subsequent patient follow-up prioritized comfort measures and arrhythmia monitoring.

## Discussion

HCM encompasses various phenotypical variants, with the predominant form being asymmetric septal hypertrophy. Other classifications include concentric, reverse septal, neutral, and apical forms [6,7]. ApHCM generally constitutes about 10% of all HCM forms, although its incidence may vary among different ethnic groups [7]. While most HCM variants share management strategies, ApHCM is notably distinct. It is more sporadic and less frequently associated with sarcomere mutations, and its risk factors for sudden cardiac death (SCD) differ from those of classic HCM [6,8].

Consistent with classic HCM, genetic mutations observed in ApHCM are primarily sarcomeric and exhibit an autosomal dominant inheritance pattern. These are modulated by environmental and ethnic determinants [6,9]. Up to 30% of cases have identifiable pathogenic gene mutations, most commonly associated with MYBPC3 and MYH7, and frequently present a familial history of HCM [7-10]. Currently, there are no established ApHCM-specific guidelines for diagnosis, family screening, or risk stratification. Both the American College of Cardiology Foundation/American Heart Association and the European Society of Cardiology lack ApHCM-specific recommendations in their latest guidelines for HCM [11,12].

ApHCM is characterized by hypertrophy of the apical segments of the LV, leading to a distinct "spade-shaped" cavity during diastole. ECG patterns often show deep negative T-waves in precordial and inferolateral leads, accompanied by voltage criteria suggestive of left ventricular hypertrophy [2,3,6-10]. Whether assessed by TTE or cMRI, diagnostic imaging frequently reveals characteristic LV apical hypertrophy [2,3,6-10]. This is quantified by an apical wall thickness of ≥15 mm and a maximal apical-to-posterior wall thickness ratio of ≥1.5 [8].

In ApHCM patients, LVOT obstruction due to anterior systolic motion of the anterior mitral valve leaflet is typically absent. However, midventricular obstruction and cavity obliteration during systole may occur [6]. Apical systolic cavity obliteration predominantly manifests in pure ApHCM but may also occur in less common variants [6]. The extent of obliteration can be quantified through the ratio of apical obliteration end-systolic length to LV cavity end-systolic length [13]. A systolic obliteration-to-cavity ratio >0.5 has been associated with an increased incidence of atrial fibrillation, stroke, heart failure, and cardiovascular mortality [13,14]. Apical aneurysms are detected in approximately 2% of all HCM patients, but in about 15% of those diagnosed with ApHCM, posing an increased risk of thrombus formation at the apex and potentially elevating the risk of thromboembolic strokes [15,16].

TTE serves as a reliable initial diagnostic tool, allowing visualization of apical hypertrophy and differentiation between pure and mixed forms of ApHCM [6]. It also identifies other prognostic factors, such as apical aneurysms and midventricular obstruction. Nevertheless, early ApHCM phenotypes may be missed by TTE [6,15,17]. When deep T-wave inversion is evident on ECG but TTE results are inconclusive, supplementary imaging like cMRI is strongly advised for its enhanced diagnostic sensitivity [17,18]. Late gadolinium enhancement (LGE) is commonly observed in HCM patients, and its prevalence and extent correlate with the severity of hypertrophy and increased propensities for heart failure and SCD [18]. In ApHCM, LGE patterns predominantly manifest in the apical and sub-endocardial regions, which are uncommon in other HCM variants [18,19].

Therapeutic options for symptomatic ApHCM are limited. Patients with LV mid-cavity obstruction are often treated with high-dose beta-blockers, diltiazem, or verapamil [11,12]. However, responses can be variable and limited. Small LV apical aneurysms typically do not require treatment, but if thrombi form, long-term oral anticoagulation is advised [11,12]. Prophylactic implantable cardioverter-defibrillator (ICD) placement is recommended for those at increased SCD risk [11,12]. Patients should be informed about exercise restrictions, and those without a history of ventricular fibrillation but at risk of SCD should undergo standardized evaluations to estimate their five-year SCD risk using the HCM risk-SCD model by the European Society of Cardiology [12]. Some patients with refractory symptoms can be considered for OHT [11,12].

Left ventricular hypertrophy in cardiac transplants may arise from multiple mechanisms, such as increased afterload due to systemic hypertension, direct myocyte proliferation from immunosuppression, or chronic inflammation [20]. Systemic hypertension is reported in about 75% of transplant recipients during the first year of post-transplantation, with a higher prevalence when using calcineurin inhibitors [21,22]. Chronic inflammatory states, especially persistent intracardiac tumor necrosis factor-alpha expression, can also induce allograft hypertrophy independent of hypertension [23]. Calcineurin inhibitors might directly cause hypertrophy by stimulating profibrotic signaling pathways [24]. However, ApHCM has not been associated with post-transplant etiologies of hypertension.

The emergence of an ApHCM phenotype in a transplanted heart suggests a genetic susceptibility to this form of HCM in the donor heart [5,25]. Current protocols do not mandate genetic screening for HCM among potential donors in the absence of a known familial history. This case report presents a rare occurrence of ApHCM in a transplanted heart, likely due to the young donor's genetic predisposition. The condition appears to be unrelated to long-term immunosuppressive therapy or hypertension. The patient was administered maximum tolerable doses of beta-blockers, resulting in modest improvement. He demonstrated a low risk for SCD, lacked an ICD, and showed no evidence of atrial fibrillation or other arrhythmias upon ambulatory monitoring. As ApHCM progresses, the development of apical aneurysms is a possibility. For some individuals, the only curative option is heart transplantation, for which this patient was ineligible. Emerging treatments like mavacampten have shown promise for HCM patients without LVOT obstruction, but their efficacy in ApHCM cases remains under-studied [26].

## Conclusions

Left ventricular ApHCM is a rare variant of HCM characterized by localized hypertrophy of the apical segment of the LV. Due to its rarity, left ventricular ApHCM has been less extensively studied than other forms of HCM. The presented case of a heart transplant recipient diagnosed with left ventricular ApHCM after 11 years post-transplant adds to the unique nature of this condition. Long-term outcomes and prognostic factors for left ventricular ApHCM in heart transplant patients remain to be elucidated. Given the scarcity of cases and limited knowledge about the long-term outcomes of left ventricular ApHCM, continued research efforts are warranted to enhance our comprehension of its pathophysiology, natural history, and optimal therapeutic approaches. A deeper understanding of left ventricular ApHCM can potentially improve early detection, risk stratification, and patient management, ultimately leading to better clinical outcomes for affected individuals.
